# Tribute to the Late Dr. Charles D. Hufford

**DOI:** 10.3390/molecules24030588

**Published:** 2019-02-07

**Authors:** Alice M. Clark

**Affiliations:** Department of BioMolecular Sciences, Division of Pharmacognosy; School of Pharmacy, The University of Mississippi, Oxford, MS 38677, USA; amclark@olemiss.edu


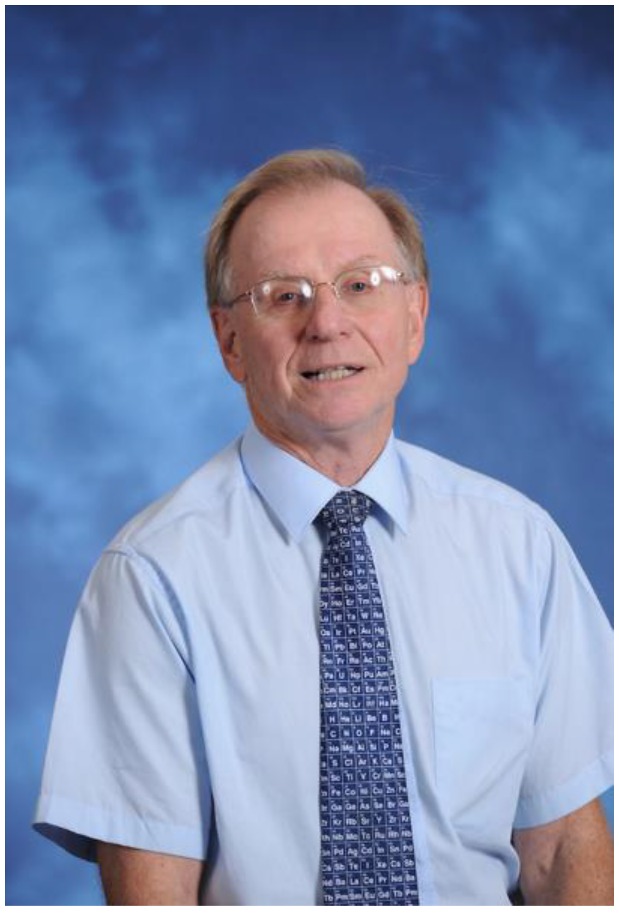


This Special Issue is dedicated to the late Dr. Charles (Charlie) D. Hufford, former Professor of Pharmacognosy and Associate Dean for Research and Graduate Studies at the University of Mississippi. Dr. Hufford passed away, May 15, 2017 at the age of 72. 

Charlie was born in the small community of Sycamore, Ohio in 1944 to Charles and Magdalena Hufford. His father, Charley, was an avid and skilled amateur botanist, teaching Charlie about the flora of the northern U.S. and Canada as they hunted and fished the areas. Charlie’s love of botany, developed as a child, eventually led him to a lifetime of work devoted to understanding the chemistry and biology of plants. After graduating from Mohawk High School in 1962, he enrolled at The Ohio State University (OSU), where he found both his career and his lifelong devotion to Buckeye sports! He graduated with a pharmacy degree from The Ohio State University in 1967. 

While at OSU, he had the good fortune to meet Jack Beal, who recognized his talent for pharmacognosy and encouraged him to pursue graduate studies. Charlie obtained his Ph.D. in pharmacognosy in 1972 under the direction of Ray Doskotch. While pursuing his graduate studies, he also served as a pharmacist in the Air Force Reserve. It was during his graduate studies that Charlie developed his passion for NMR spectroscopy, eventually becoming a leader in the application and interpretation of NMR to the structure elucidation of novel natural products. 

He joined the faculty of the University of Mississippi (UM) School of Pharmacy as an assistant professor in the summer of 1972. Throughout his career he served the school in several roles, including two terms of service as department chair before becoming the school’s first associate dean for research and graduate studies in 1995. For 42 years he was a tireless advocate for the School of Pharmacy graduate students, faculty, and staff. He originated important research programs that continue today, was one of the principal investigators for the long-running NIH-funded antifungal research program, and was the singular driving force behind building the school’s capacity in NMR spectroscopy. He retired from the university in January 2015 to devote his time to his two highest priorities: grandsons and bowling! 

Charlie was a quiet leader who worked out of the spotlight to support the careers of countless students and colleagues. Throughout his own career, he collaborated with many other scientists to identify hundreds of natural products representing a wide array of chemotypes and an impressive range of bioactivities. He was also a pioneer in the use of microorganisms to predict mammalian metabolism of drugs, and was instrumental in advocating that the University of Mississippi School of Pharmacy incorporate the understanding of dietary supplements into its research and educational initiatives several years before the Dietary Supplement Health and Education Act in 1994.

Charlie’s greatest professional achievement was his role in developing scores of scientists, educators, pharmacists, and leaders. He was a patient and gifted mentor and teacher who set many graduate students on their way to fulfilling and influential lives in academia, industry, government, and nonprofit organizations throughout the world. His devotion to graduate education in pharmacognosy continues through the Charles D. Hufford Graduate Student Fellowship Endowment at The University of Mississippi, which he established when he retired in order to support in perpetuity graduate education in pharmacognosy. 

Charlie received many honors and awards for both his professional and personal accomplishments, including the 1994 UM School of Pharmacy Outstanding Researcher of the Year and the 1995 OSU College of Pharmacy Jack Beal Award. He was an elected Fellow of the American Association of Pharmaceutical Scientists, and served in every leadership position of the American Society of Pharmacognosy. 

He also had a passion and skill for bowling—he bowled more than 30 perfect 300 games over his bowling career, the latest on December 14, 2016. In that sphere as well, he was widely known and admired as a mentor, coach, teammate, and friend to all.

I had the unique privilege of being partner to one of the greatest minds and most generous hearts in science—Charlie always gave more than he got. Like so many others, I was the beneficiary of his skill and expertise, his patient and methodical approach to our work, and his unwavering commitment to the highest standards. 

It is fitting that this Special Issue comprises papers from former students, collaborators, and colleagues throughout the world who have utilized an impressive array of techniques to probe every possible natural source to identify a variety of chemotypes with a wide spectrum of biological activities—with some new microbial transformations and insights into dietary supplements as well. It is an appropriate reflection of Charlie’s career that spanned 40+ years, during which he contributed to both our scientific knowledge and the development of scientists throughout the world.

